# A population-based study of incidence trends of head and neck epithelial cancers in northeastern Spain, 1994–2018

**DOI:** 10.1007/s12094-025-03855-8

**Published:** 2025-02-19

**Authors:** Jordi Rubió-Casadevall, Jan Trallero, Carla Calvo, Montse Puigdemont, Marià Carulla, Arantza Sanvisens, Alberto Ameijide, Anna Vidal, Clàudia Pla, Jordi Marruecos, Rafael Marcos-Gragera, Jaume Galceran

**Affiliations:** 1 Medical Oncology Department, Catalan Institute of Oncology-Girona (ICO), Hospital Josep Trueta, Girona, Spain; 2 Descriptive Epidemiology, Genetics and Cancer Prevention Group, Girona Biomedical Research Institute of Girona (IDIBGI-CERCA), Girona, Spain; 3 School of Medicine, University of Girona (UdG), Girona, Spain; 4 Epidemiology Unit and Girona Cancer Registry, Oncology Coordination Plan, Department of Health Government of Catalonia, Catalan Institute of Oncology, Girona, Spain; 5 Josep Carreras Leukaemia Research Institute, Girona, Spain; 6 Cancer Registry of Tarragona, Cancer Epidemiology and Prevention Service, Hospital Universitari Sant Joan de Reus, Reus, Spain; 7 Oncological, Epidemiological, Translational and Clinical Research Group (GIOTEC), Institut d’Investigació Sanitària Pere Virgili (IISPV), Reus, Spain; 8 Radiotherapy Oncology Department, Catalan Institute of Oncology, Hospital Josep Trueta, Girona, Spain

**Keywords:** Epidemiology, Head and neck cancer, Incidence, Trends

## Abstract

**Background:**

Head and neck cancer (HNC) is the seventh most common cancer worldwide. Incidence rates of these tumors vary between countries and change over time depending on the prevalence of risk factors such as tobacco and alcohol consumption, betel nut chewing habits or human papillomavirus infection. This makes it necessary to periodically study HNC incidence trends in each geographic area.

**Methods:**

To determine trends in the incidence of HNC, all cancer cases diagnosed in Girona and Tarragona (northeastern Spain) between 1994 and 2018, except mesenchymal and hematological neoplasms, were included. Crude and standardized incidence rates and the annual percentage change (APC) were calculated.

**Results:**

We identify 7,966 cases of HNC, 83.7% in men. A significant decrease in incidence, with an APC of  – 1.83, was observed in all HNC as a whole and in cancers of the lip (APC =  – 5.34), salivary glands (APC =  – 2.22), nasopharynx (APC =  – 2.01), hypopharynx (APC =  – 3.15), and larynx (APC =  – 1.97). In men, a significant decline in incidence was observed in overall HNC and in cancers of the lip, oral cavity, salivary glands, nasopharynx, hypopharynx, and larynx. In women, a significant increase was identified in overall HNC and in cancers of oral cavity, oropharynx, hypopharynx, and larynx.

**Conclusion:**

A decline in the overall incidence of HNC has been observed in this area of southern Europe, mainly based on a decrease in men of cancers of the lip, oral cavity, salivary glands, nasopharynx, hypopharynx, and larynx.

**Supplementary Information:**

The online version contains supplementary material available at 10.1007/s12094-025-03855-8.

## Introduction

Head and neck cancers (HNC) are the seventh most incident cancers worldwide, accounting for an estimated 947,000 cases (approximately 4.5% of all cancer diagnoses worldwide) in 2022. In addition, the number of deaths from these cancers is estimated to be more than 450,000 per year, approximately 5% of all cancer deaths [1].

Incidence rates of these cancers vary between countries depending on the prevalence of risk factors such as tobacco and alcohol consumption, betel nut chewing, genetic characteristics, or related-virus infection. Thus, nasopharyngeal cancer (NPC) has a higher incidence in North Africa and Southeast Asia [2] due to prevalence of Epstein–Barr virus (EBV) infection and genetic predisposition, and oral cancer in Southeast Asia due to betel nut chewing habit [3]. Some countries, mainly in North America and Northern Europe, have a higher number of oropharyngeal cancers (OPC) due to human papillomavirus (HPV) than others [4], and an increasing incidence has been reported in these countries [5].

HNC is more common in men than in women, with a male-to-female ratio of approximately 2:1, and in adults over 50 years of age [1]. Younger women in developed countries have experienced an increase of incidence, probably due to changes in gender-specific cultural expectations regarding tobacco and alcohol consumption, as well as an increasing burden of HPV [6].

Demographic changes and changes in carcinogen exposure over time in Spain make it necessary to determine the incidence patterns of HNC. Therefore, our aim was to determine the incidence and incidence trends of these cancers in northeastern Spain, in the provinces of Girona and Tarragona, which have a population-based cancer registry.

## Materials and methods

### Population of study

This is a population-based retrospective cohort study of HNC cases diagnosed in the provinces of Girona and Tarragona between 1994 and 2018. All cases were collected by the Girona Cancer Registry (GCR) and the Tarragona Cancer Registry (TCR) which covered a population at risk of 749,656 and 794,969 inhabitants in 2018, respectively (http://www.idescat.cat, accessed on 31 March 2024). Data collection and recording in both registries is carried out in accordance with the International Agency for Research on Cancer (IARC) guidelines and both registries are members of the International Association of Cancer Registries (IACR), the European Network of Cancer Registries (ENCR) and the Spanish Network of Cancer Registries (REDECAN).

Cases of malignant tumors diagnosed on head and neck topographies in patients residing in the areas covered by both registries and coded according to the International Classification of Diseases for Oncology, Third Revision (ICD-O-3) were included in the study [7]. Demographic (sex, age at diagnosis) and tumor (year of diagnosis, topography, histology, and behavior) information was available for each tumor case. Head and neck topographies were grouped into nine groups: lip, oral cavity, oropharynx, salivary glands, nasopharynx, hypopharynx, pharynx not otherwise specified (pharynx, NOS), nasal sinuses, and larynx. Further details on the topography codes for each group are displayed in Supplementary Table 1. Cases diagnosed in patients not residing in the provinces of Girona or Tarragona, cases of non-invasive HNC or non-clearly malignant (ICD-O-3 morphological behavior code 0, 1 or 2) and cases with ICD-O-3 morphology codes from 8800 to 9993 (corresponding to histology of lymphoma and sarcoma) were excluded.

Cases are registered tumors, so the number of cases is greater than the number of patients given the presence of multiple primary tumors.

### Statistical analysis

A descriptive analysis of the distribution of cases overall and by sex was performed as absolute and relative frequencies for qualitative variables and as mean and standard deviation for quantitative variables. Statistical comparisons between sexes were calculated using the *T* test for quantitative variables, and Chi-squared or Fisher’s exact test, for qualitative variables, as appropriate.

Incidence rates were expressed as crude incidence rates (CIR) and age-standardized incidence rates (ASIR). Crude incidence rates were calculated as the number of cancer cases divided by the population at risk multiplied by 100,000 during a specific time period to express cases per 100,000 person-years. Similarly, age-standardized incidence rates were calculated using the direct method by applying weights to each age group according to the population standard used. Age-standardized incidence rates were calculated by the direct method using the 2013 European standard population (ASIRe) and the 1960 Segi world standard population (ASIRw). The population at risk for each registry and calendar year was obtained from the Spanish National Statistics Institute.

Incidence rate trends were evaluated by period of diagnosis (1994–2001, 2002–2009, and 2010–2018) and were also modeled using Poisson generalized linear models adjusted by age. Segmented models were tested to evaluate possible point changes in the trend and the annual percentage change (APC) was estimated for each segment.

All statistical analyses were performed with R version 4.3.2.

## Results

### Gender, age, and histological characteristics

A total of 7,966 cases of HNC were identified by the GCR and TCR between 1994 and 2018, for which 83.7% of them were diagnosed in men, and the mean ± standard deviation (SD) age at diagnosis was 64.1 ± 13.0 years. The larynx was the most common subsite in both sexes as a whole and in men, while the oral cavity was the most common site in women, as shown in Table 1. In addition, Table 2 shows cases in each HNC topography stratified by sex and age. The youngest mean ± SD age at diagnosis was in nasopharyngeal cancer in both men and women (52.9 ± 15.0 and 54.2 ± 17.3 years, respectively) and the oldest was in lip cancer also for both sexes (70.8 ± 11.6 years in men and 77.7 ± 10.0 years in women).

**Table 1:** Characteristics of head and neck cancer cases diagnosed in the provinces of Girona and Tarragona, 1994–2018

	Men *N* = 6667 *n* (%)	Women *N* = 1299 *n* (%)	Total *N* = 7966 *n* (%)	*p* value [*]
*Age at diagnosis*				< 0.01
Mean ± SD	63.5 ± 12.4	67.0 ± 15.5	64.1 ± 13.0	
*Age group *(*years*)				< 0.01
0–49	892 (13.4)	179 (13.8)	1,071 (13.4)	
50–59	1,646 (24.7)	240 (18.5)	1,886 (23.7)	
60–69	1,949 (29.2)	259 (19.9)	2,208 (27.7)	
70–79	1,481 (22.2)	299 (23.0)	1,780 (22.3)	
80 +	699 (10.5)	322 (24.8)	1,021 (12.8)	
*HN cancer site*				< 0.01
Lip	823 (12.3)	149 (11.5)	972 (12.2)	
Oral cavity	1,173 (17.6)	522 (40.2)	1,695 (21.3)	
Salivary glands	178 (2.7)	156 (12.0)	334 (4.2)	
Oropharynx	991 (14.9)	159 (12.2)	1,150 (14.4)	
Nasopharynx	199 (3.0)	68 (5.2)	267 (3.4)	
Hypopharynx	591 (8.9)	32 (2.5)	623 (7.8)	
Pharynx, NOS	56 (0.8)	7 (0.5)	63 (0.8)	
Nasal sinuses	146 (2.2)	65 (5.0)	211 (2.6)	
Larynx	2,510 (37.6)	141 (10.9)	2,651 (33.3)	

*p* value [*]: independent samples *T* test for mean age at diagnosis, Chi-squared test of independence for age group, Fisher’s exact test for HN cancer sites

*HN* head and neck, *NOS* not otherwise specified, *SD* standard deviation

**Table 2:** Distribution of head and neck cancer cases by subsite, sex, and age at diagnosis in the provinces of Girona and Tarragona, 1994–2018

HN cancer site	Age at diagnosis (years) Mean ± SD	Age group (years)				
		0–49 *N* = 1071 *n* (%)	50–59 *N* = 1886 *n* (%)	60–69 *N* = 2208 *n* (%)	70–79 *N* = 1780 *n* (%)	80 + *N* = 1021 *n* (%)
*Lip*						
Men	70.8 ± 11.6	38 (4.6)	100 (12.2)	200 (24.3)	287 (34.9)	198 (24.1)
Women	77.7 ± 10.0	2 (1.3)	4 (2.7)	20 (13.4)	54 (36.2)	69 (46.3)
*Oral cavity*						
Men	62.3 ± 12.8	184 (15.7)	294 (25.1)	344 (29.3)	251 (21.4)	100 (8.5)
Women	68.8 ± 15.3	66 (12.6)	81 (15.5)	99 (19.0)	121 (23.2)	155 (29.7)
*Salivary glands*						
Men	70.2 ± 15.4	16 (9.0)	29 (16.3)	27 (15.2)	51 (28.7)	55 (30.9)
Women	67.3 ± 17.8	25 (16.0)	26 (16.7)	20 (12.8)	35 (22.4)	50 (32.1)
*Oropharynx*						
Men	60.5 ± 11.0	160 (16.2)	326 (32.9)	285 (28.8)	172 (17.4)	48 (4.8)
Women	61.5 ± 13.1	23 (14.5)	52 (32.7)	45 (28.3)	27 (17.0)	12 (7.6)
*Nasopharynx*						
Men	52.9 ± 15.0	94 (47.2)	41 (20.6)	34 (17.1)	21 (10.6)	9 (4.5)
Women	54.2 ± 17.3	26 (38.2)	15 (22.1)	13 (19.1)	9 (13.2)	5 (7.4)
*Hypopharynx*						
Men	61.2 ± 10.8	82 (13.9)	185 (31.3)	201 (34.0)	86 (14.6)	37 (6.3)
Women	61.0 ± 13.6	7 (21.9)	9 (28.1)	9 (28.1)	4 (12.5)	3 (9.4)
*Pharynx, NOS*						
Men	61.4 ± 12.6	8 (14.3)	20 (35.7)	13 (23.2)	10 (17.9)	5 (8.9)
Women	58.1 ± 12.6	2 (28.6)	3 (42.9)	0 (0.0)	2 (28.6)	0 (0.0)
*Nasal sinuses*						
Men	64.9 ± 14.3	27 (18.5)	20 (13.7)	37 (25.3)	38 (26.0)	24 (16.4)
Women	70.0 ± 14.1	7 (10.8)	7 (10.8)	12 (18.5)	20 (30.8)	19 (29.2)
*Larynx*						
Men	63.7 ± 11.4	283 (11.3)	631 (25.1)	808 (32.2)	565 (22.5)	223 (8.9)
Women	61.8 ± 11.4	21 (14.9)	43 (30.5)	41 (29.1)	27 (19.2)	9 (6.4)

*HN* head and neck, *SD* standard deviation, *NOS* not otherwise specified

Histological variants of squamous cell neoplasms (ICD-O-3 morphology codes 8050–8086) were the most prevalent in most sites. They accounted for 98.2% in lip cancer, 92.9% in oral cancer, 92.4% in OPC, including those histologies of squamous cell carcinoma (SCC) HPV-related, 92.3% in hypopharyngeal cancer, 55.9% in sinonasal cancer, and 92.4% in laryngeal cancer. In NPC, it constituted 67.8% of all cases but it should be noted that some EBV-related carcinomas were coded as undifferentiated carcinoma (code 8020). In the salivary gland, SCC was 24.0%, followed by mucoepidermoid carcinoma, which accounted for 15.6%. The evolution in the definition of ICD-O-3 morphology codes, the incorporation of new codes, especially those related to viral tumor etiology, and the lack of precision in some diagnoses, especially the oldest ones, must be considered in the interpretation of the distribution by histology. Supplementary Table 2 shows the distribution of cases according to the histological code for each site.

### Incidence

**Fig. 1:**
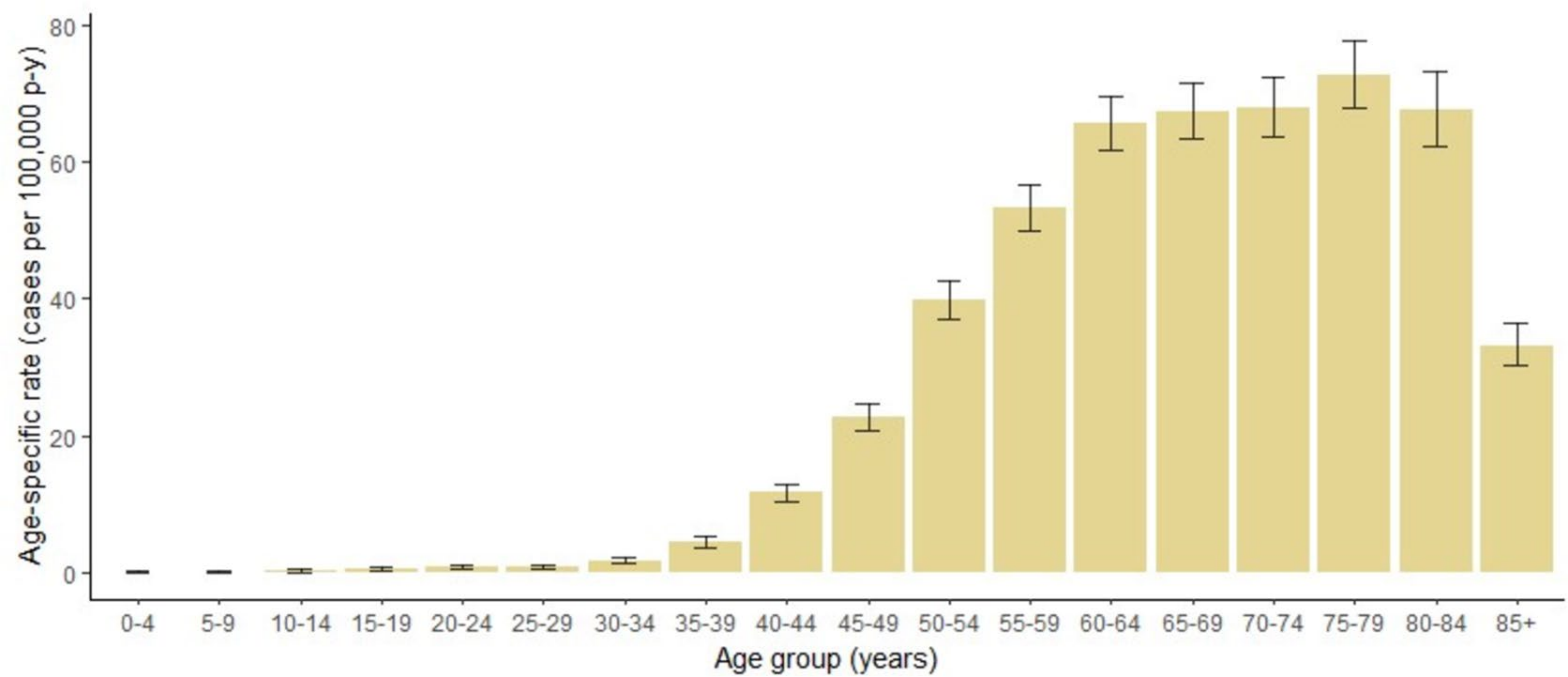
Age-specific rates of head and neck cancers in the provinces of Girona and Tarragona, 1994–2018

Age-specific rates of HNC as a whole are displayed in Fig. 1. The age group with the highest rate of HNC was 75–79 years. CIR, ASIRe and ASIRw of HNC as a whole and by subsite stratified by sex are presented in Table 3. The overall CIR was 23.04 cases per 100,000 person-years (95% confidence interval (CI) 22.53, 23.55), and the ASIRe, 25.62 cases per 100,000 person-years (95% CI 25.06, 26.19). Sex differences were observed, as the incidence in men was six times higher compared to women. Men showed an ASIRe of 45.28 (95%CI 44.19, 46.38) cases per 100,000 person-years compared with an ASIRe of 7.55 (95%CI 7.13, 7.98) in women. The largest gender difference was seen in hypopharyngeal cancer which is 21 times more incident in men than in women.

**Table 3:** Incidence rates of head and neck cancer sites by sex in the provinces of Girona and Tarragona, 1994–2018

HN cancer site	N	CIR (95%CI)	ASIR_E_ (95%CI)	ASIR_W_ (95%CI)
*Overall*				
Both	7,966	23.04 (22.53–23.55)	25.62 (25.06–26.19)	13.93 (13.60–14.26)
Men	6,667	38.65 (37.73;39–59)	45.28 (44.19–46.38)	24.27 (23.66–24.89)
Women	1,299	7.50 (7.09;7.92)	7.55 (7.13–7.98)	3.93 (3.69–4.18)
*Lip*				
Both	972	2.81 (2.64–2.99)	3.07 (2.88–3.27)	1.29 (1.20–1.39)
Men	823	4.77 (4.45;5.11)	5.78 (5.39–6.19)	2.42 (2.24–2.60)
Women	149	0.86 (0.73–1.01)	0.79 (0.66–0.93)	0.27 (0.22–0.33)
*Oral cavity*				
Both	1,695	4.90 (4.67–5.14)	5.38 (5.13–5.65)	2.91 (2.76–3.06)
Men	1,173	6.80 (6.42–7.20)	7.91 (7.46–8.37)	4.36 (4.11–4.63)
Women	522	3.01 (2.76–3.28)	2.97 (2.71–3.24)	1.47 (1.32–1.62)
*Salivary glands*				
Both	334	0.97 (0.87–1.08)	1.01 (0.90–1.13)	0.48 (0.42–0.54)
Men	178	1.03 (0.89–1.20)	1.21 (1.04–1.41)	0.52 (0.43–0.61)
Women	156	0.90 (0.76–1.05)	0.87 (0.74–1.03)	0.45 (0.37–0.55)
*Oropharynx*				
Both	1,150	3.33 (3.14–3.52)	3.76 (3.54–3.98)	2.23 (2.09–2.36)
Men	991	5.74 (5.39–6.11)	6.65 (6.24–7.08)	3.87 (3.63–4.13)
Women	159	0.92 (0.78–1.07)	1.00 (0.85–1.18)	0.60 (0.51–0.71)
*Nasopharynx*				
Both	267	0.77 (0.68–0.87)	0.81 (0.71–0.91)	0.57 (0.50–0.65)
Men	199	1.15 (1.00–1.33)	1.21 (1.05–1.40)	0.85 (0.73–0.98)
Women	68	0.39 (0.30–0.50)	0.41 (0.32–0.52)	0.29 (0.22–0.38)
*Hypopharynx*				
Both	623	1.80 (1.66–1.95)	2.04 (1.88–2.21)	1.21 (1.11–1.31)
Men	591	3.43 (3.16–3.71)	3.98 (3.67–4.32)	2.32 (2.13–2.52)
Women	32	0.18 (0.13–0.26)	0.19 (0.13–0.28)	0.12 (0.08–0.17)
*Pharynx NOS*				
Both	63	0.18 (0.14–0.23)	0.20 (0.15–0.26)	0.12 (0.09–0.15)
Men	56	0.32 (0.25–0.42)	0.37 (0.28–0.48)	0.21 (0.16–0.28)
Women	7	0.04 (0.02–0.08)	0.04 (0.02–0.09)	0.03 (0.01–0.06)
*Nasal sinuses*				
Both	211	0.61 (0.53–0.70)	0.66 (0.57–0.76)	0.33 (0.28–0.38)
Men	146	0.85 (0.71–1.00)	0.98 (0.83–1.16)	0.49 (0.41–0.59)
Women	65	0.38 (0.29–0.48)	0.37 (0.28–0.47)	0.17 (0.12–0.22)
*Larynx*				
Both	2,651	7.67 (7.38–7.96)	8.69 (8.36–9.02)	4.80 (4.61–4.99)
Men	2,510	14.55 (13.99–15.13)	17.17 (16.50–17.86)	9.23 (8.85–9.61)
Women	141	0.81 (0.68–0.96)	0.90 (0.75–1.06)	0.53 (0.44–0.63)

Incidence rates expressed as cases per 100,000 person-years. *ASIR*_*E*_ age-standardized incidence rate using the 2013 European standard population, *ASIR*_*W*_ age-standardized incidence rate using the 1960 World standard population, *CI* confidence interval, *CIR* crude incidence rate, *NOS* not otherwise specified

Laryngeal cancer was the site with the highest ASIRe, that varies from 10.76 (95%CI 10.04, 11.51) in 1994–2001 to 7.72 (95%CI 7.24, 8.22) cases per 100,000 person-years in 2010–2018, both sexes included. Incidence evolution by period of diagnosis for all HNC and HNC subsites are displayed in Table 4.

**Table 4:** Incidence rates of head and neck cancer sites by period of diagnosis in the provinces of Girona and Tarragona, 1994–2018

HN Cancer site	N	CIR (95%CI)	ASIR_E_ (95%CI)	ASIR_W_ (95%CI)
*Overall*				
1994–2001	2,399	26.55 (25.50–27.63)	30.73 (29.51–32.00)	17.04 (16.32–17.78)
2002–2009	2,545	22.57 (21.70–23.46)	25.87 (24.87–26.91)	14.15 (13.56–14.75)
2010–2018	3,022	21.19 (20.44–21.96)	22.75 (21.93–23.59)	12.07 (11.61–12.55)
*Lip*				
1994–2001	369	4.08 (3.68–4.52)	4.78 (4.30–5.30)	2.13 (1.90–2.38)
2002–2009	341	3.02 (2.71–3.36)	3.39 (3.04–3.78)	1.40 (1.23–1.57)
2010–2018	262	1.84 (1.62–2.07)	1.85 (1.63–2.10)	0.71 (0.61–0.82)
*Oral cavity*				
1994–2001	447	4.95 (4.50–5.43)	5.70 (5.18–6.26)	3.18 (2.87–3.51)
2002–2009	537	4.76 (4.37–5.18)	5.38 (4.93–5.86)	2.93 (2.67–3.21)
2010–2018	711	4.98 (4.62–5.36)	5.26 (4.87–5.67)	2.75 (2.53–2.98)
*Salivary glands*				
1994–2001	98	1.08 (0.88–1.32)	1.28 (1.04–1.56)	0.56 (0.44–0.70)
2002–2009	111	0.98 (0.81–1.19)	1.04 (0.86–1.26)	0.50 (0.40–0.62)
2010–2018	125	0.88 (0.73–1.04)	0.89 (0.73–1.06)	0.44 (0.34–0.54)
*Oropharynx*				
1994–2001	280	3.10 (2.75–3.48)	3.57 (3.16–4.02)	2.20 (1.94–2.48)
2002–2009	367	3.25 (2.93–3.60)	3.82 (3.44–4.23)	2.25 (2.02–2.50)
2010–2018	503	3.53 (3.22–3.85)	3.88 (3.55–4.24)	2.26 (2.06–2.48)
*Nasopharynx*				
1994–2001	83	0.92 (0.73–1.14)	1.00 (0.80–1.24)	0.70 (0.55–0.87)
2002–2009	84	0.74 (0.59–0.92)	0.81 (0.65–1.01)	0.56 (0.44–0.71)
2010–2018	100	0.70 (0.57–0.85)	0.71 (0.57–0.86)	0.52 (0.42–0.65)
*Hypopharynx*				
1994–2001	206	2.28 (1.98–2.61)	2.65 (2.30–3.04)	1.65 (1.42–1.90)
2002–2009	213	1.89 (1.64–2.16)	2.23 (1.94–2.55)	1.34 (1.16–1.54)
2010–2018	204	1.43 (1.24–1.64)	1.56 (1.36–1.80)	0.86 (0.74–1.00)
*Pharynx NOS*				
1994–2001	19	0.21 (0.13–0.33)	0.24 (0.15–0.38)	0.16 (0.10–0.25)
2002–2009	19	0.17 (0.10–0.26)	0.18 (0.11–0.29)	0.11 (0.07–0.18)
2010–2018	25	0.18 (0.11–0.26)	0.19 (0.12–0.29)	0.10 (0.06–0.15)
*Nasal sinuses*				
1994–2001	59	0.65 (0.50–0.84)	0.75 (0.57–0.97)	0.37 (0.28–0.49)
2002–2009	59	0.52 (0.40–0.67)	0.55 (0.42–0.72)	0.28 (0.21–0.37)
2010–2018	93	0.65 (0.53–0.80)	0.69 (0.55–0.85)	0.34 (0.26–0.42)
*Larynx*				
1994–2001	838	9.27 (8.66–9.92)	10.76 (10.04–11.51)	6.09 (5.67–6.54)
2002–2009	814	7.22 (6.73–7.73)	8.47 (7.89–9.08)	4.77 (4.43–5.13)
2010–2018	999	7.00 (6.58–7.45)	7.72 (7.24–8.22)	4.09 (3.82–4.36)

Incidence rates expressed as cases per 100,000 person-years. *ASIR*_*E*_ age-standardized incidence rate using the 2013 European standard population, *ASIR*_*W*_ age-standardized incidence rate using the 1960 World standard population, *CI* confidence interval, *CIR* crude incidence rate, *NOS* not otherwise specified

### Trends in incidence

The incidence of HNC decreased significantly for both sexes, with an APC of  – 1.83 (95% CI  – 2.14,  – 1.52). This decrease is attributed to the statistically significant decrease observed in men, with an APC of  – 2.63 (95% CI  – 2.96,  – 2.29), in contrast to women, who have significantly increased their incidence rates, with an APC of 1.77 (95% CI 0.97, 2.96).

**Fig. 2:**
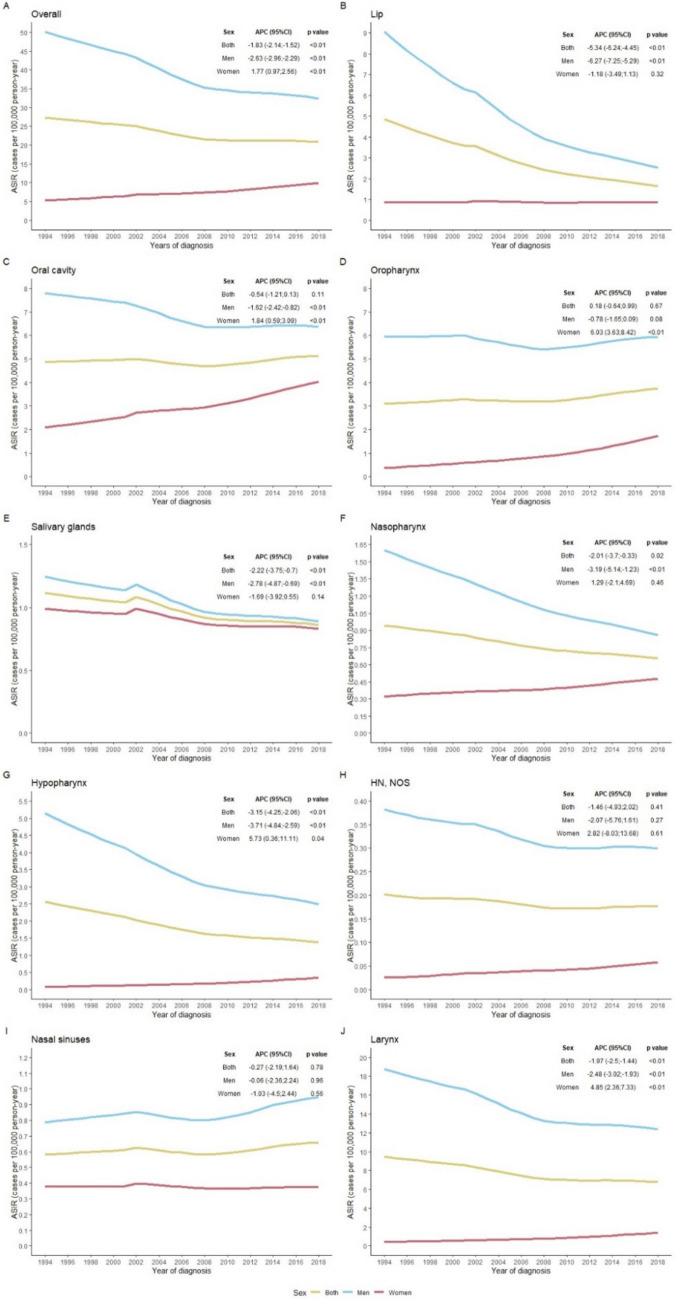
Incidence trends and annual percentage change (APC) of head and neck cancers as a whole and by subsite and sex in the provinces of Girona and Tarragona, 1994–2018

The overall incidence decreased significantly throughout the study period for cancers of the lip, salivary glands, nasopharynx, hypopharynx, and larynx, with an APC of  – 5.34,  – 2.22,  – 2.01,  – 3.15, and  – 1.97, respectively. The remaining sites showed a stable incidence rate with no increase in incidence at any of the subsites (Fig. 2).

In men, a significant downward trend was observed in cancers of the lip, oral cavity, salivary glands, nasopharynx, hypopharynx, and larynx, with an APC of  – 6.27,  – 1.62,  – 2.78,  – 3.19,  – 3.71, and  – 2.48, respectively. No cancer site showed an increased incidence in men. In women, cancer of the oral cavity, oropharynx, hypopharynx, and larynx significantly increased, with an APC of 1.84, 6.03, 5.73 and 4.85, respectively. The remaining sites showed stability in the incidence throughout the study period. Figure 2 shows the APC of HNC incidence as a whole and for specific topographies.

In this study of trends in incidence, no change points were observed.

## Discussion

In this study, we analyzed the incidence and incidence trends of different sites of HNC in both men and women. The grouping of tumor locations was based on clinical criteria and may differ from that of other epidemiological studies.

The most incident cancer site in the head and neck area varies worldwide. In Spain and Southern Europe, the larynx predominates as the main site of HNC [1]. In central or northern Europe, the most incident sites are the oropharynx, as in the United States (US), and the oral cavity [8].

Our results show a statistically significant decrease in the overall HNC incidence, driven primarily by a decrease in the incidence among men. On the assumption that our population has the same evolution of smoking habits as that of Spain as a whole, we consider that the decrease in smoking among men, who have traditionally been the predominant gender in HNC, is the main explanation for the decline in incidence. In contrast, we observed an upward trend among women, as also reported by other studies, often attributed to the fact that women started smoking later than men [9]. In Spain, as evidenced by the Spanish National Health Survey, there has been a reduction in tobacco consumption, with the prevalence of daily smoking among Spaniards decreasing from 34% in 1995 to 24% in 2011 [10].

### Oropharyngeal cancer

As has happened in other Western and Northern European countries, we expected an increase in the OPC, which has not been observed, both for both sexes as a whole and for men [5]. This could be explained by the decline in tobacco and alcohol consumption in men, while the increase in cases due to HPV is not strong enough to change the trend, given that southern Europe is an area with a lower burden of HPV-related cases [11]. Although it is the site that increased the most in women, a stabilization of incidence was observed for both sexes due to the decrease observed in men. Sexual behavior related to HPV infection varies greatly from one region to another, with proportions of individuals reporting oral sex exceeding 65% in the USA compared to less than 20% in countries in southern Europe countries such as Spain [12]. That could explain the substantial increase in OPC in the USA in recent years compared to our area [13]. We could not directly evaluate the association between OPC cases and HPV infection-related causality because information on HPV DNA in tumor tissue of all cases was not available. Previously, we reported an increase of HPV-positive OPC in the GCR area [14], in a study in which p16 immunostaining was performed in almost all cases. Nevertheless, we expect this trend to increase in the coming decades due to the increase in HPV-related OPC.

### Lip cancer

The marked decrease in incidence of lip cancer observed in men and the stable trend observed in women is consistent with those observed in Austria, Denmark and Poland, Australia, and the USA [6,15]. The main risk factor associated with lip cancer is chronic sun exposure [16] and the decline in incidence could be attributed to a decrease in the number of working hours in outdoor occupations. However, the evidence of the association between lip and oral cancer to tobacco smoking, especially pipe smoking, may explain the observed trends, given the decreasing prevalence of smoking in recent decades. In our study, due to the low number of cases of this subsite, we could not discriminate if the incidence decreases were stronger for the lower lip, which is more closely related to sun exposure and pipe smoking.

### Oral cancer

Oral cancer incidence decreases in males and increases in females, both significantly. This is consistent with the trends in tobacco consumption, emphasizing the causal role of smoking in the occurrence of oral cancers. Decreasing trends in males had also been described in the USA and in several European countries, including Spain, Italy, France, and Croatia [6]. This is also consistent with our findings. Conversely, among women, rising rates are observed in several various populations, notably in Spain, Poland, Denmark, Slovenia, Estonia, Slovakia, the UK, and Japan. The highest incidence rate is in India, where betel quid chewing has been reported to increase the risk of oral cancer, independently of other tobacco and alcohol consumption [17].

### Laryngeal and hypopharyngeal cancer

In our study, a downward trend in the incidence in hypopharyngeal and laryngeal cancer was observed among men, while it increased in women, suggesting, as happened in other sites, that smoking cessation, which is more common in men, is the main factor for this reduction. The incorporation of women into the smoking habit is not yet large enough to prevent both sexes from experiencing a decreasing incidence rate. Similar trend, especially in laryngeal cancer, has been described in other European countries [18,19], worldwide [20], and previously in Spain [21].

### Sinonasal cancer

Sinonasal cancer, especially adenocarcinoma, has been strongly associated with long-term occupational exposure to hardwood or leather dust [22], although tobacco also plays a biological role. We identified a slightly but non-significant downward trend in sinonasal cancer in each and in both sexes together. This is in line with findings in other European countries [23,24], where this decrease is explained by the lower number of workers in high-risk occupations and the preventive measures taken. A trend analysis of the incidence of sinonasal cancer using data from Surveillance Epidemiology and End Results (SEER) also obtained a slight decrease, although it did not reach statistical significance [25]. The variability of histology in this location, SCC, adenocarcinoma, melanoma, and others. makes it interesting to perform a study focused on them, but as it is a rare tumor, it is difficult to have enough cases to be able to draw conclusions from a subgroup analysis.

### Nasopharyngeal cancer

We found a non-significant decreasing trend in the incidence of NPC, due to the decrease in incidence among men, in contrast to a slightly increasing trend observed in women. We expected an increase in our region due to the migratory phenomenon from countries with a high prevalence of NPC, such as Southeast Asia and North Africa. Spain has received a high immigration from North Africa, especially of Morocco, which corresponded to 12.6% of the immigrant population in 2015 [26]. In our series, 15.7% of cases were in patients born in one of these two areas, almost all from Morocco, which has an ASIRw of 2.4 cases per 100.000 person-years (GLOBOCAN, https://gco.iarc.fr/today/en/dataviz/maps-heatmap?mode=population&cancers=4). The geographic distribution of NPC suggests an association with genetic susceptibility [27], environmental factors, dietary habits, and lifestyle in addition to EBV virus infection [28]. Therefore, we anticipate that the trend in NPC incidence will change in the coming decades, due to the rising and aging of the migrant population.

### Cancer of salivary glands

The incidence of salivary gland cancers exhibits a statistically significant trend toward reduction, particularly among men. These results lead us to consider that it may be attributed to improvement in diagnosis and consequently in registration, as SCC of the skin can metastasize to the parotid gland and, in the past, skin SCC may have been underreported, which may have led to the loss of identification of the primary tumor. SCC represents 1% of salivary neoplasms and its coding is restricted to the major ones [29]. In our series, SCC represented almost 24% of cases (Supplementary Table 2), in greater proportion in the first years of the period. This can be a source of bias. However, a decrease in incidence has also been described by other cancer registries in Spain [30].

### Pharyngeal NOS cancer

Regarding pharyngeal cancer NOS, no statistically significant changes were observed in either men or women during the study period. We expected a decline in the overall incidence trend of pharyngeal cancer NOS due to improved diagnostic techniques in the later dates of the study calendar, which should have led to greater accuracy in the diagnosis and classification of the site. However, the low number of cases at this site, 0.8% of the total series, shows that cases are very accurately recorded.

Although cases are reviewed before registration, a possible topographical misclassification, which depends on the clinician making the diagnosis, could slightly vary the results and be a source of bias, even more so considering that HNC has different ICD-O site codes so close together in a small anatomical area. Therefore, we consider the grouping of locations on a clinical basis to be very relevant and necessary for future comparisons and studies. Nevertheless, the epidemiological view is a strength of this study.

Even though we know that smoking has decreased, and that HPV burden is low as an etiology of neoplasia in Spain, it is more difficult to interpret the effect of alcohol consumption in the cohort that develops cancer in our period, although it is known that its consumption increases in our country [31].

## Conclusion

In the period 1994–2018, the incidence of cancers of the HN, lip, salivary glands, nasopharynx, hypopharynx, and larynx decreased in northeast of Spain, due to the decrease in HNC incidence in men. However, women showed an increased incidence of some of these cancers. The differences between the sexes may be mainly due to changes in the smoking habits of men and women.

## Supplementary Information

Below is the link to the electronic supplementary material.

Supplementary file1 (DOCX 18 KB)

## Data Availability

Data is available at Girona's and Tarragona's Cancer Registry, but is no available to create a link.
