# Supplementary material for: A population-based study of incidence trends of head and neck epithelial cancers in northeastern Spain, 1994–2018
Source: Clin Transl Oncol. 2025 Feb 19;27(8):3410–20. doi: 10.1007/s12094-025-03855-8 (PMC12259777; doi:10.1007/s12094-025-03855-8)
Supplement: Supplementary file 1 — Supplementary file1 (DOCX 18 KB) [file 12094_2025_3855_MOESM1_ESM.docx]

# Supplementary material

**Supplementary Table 1:** International Classification of Diseases for Oncology, Third Edition (ICDO-3) topography codes by head and neck cancer sites.

| Head and neck cancer site | ICD-O-3 Topography codes |
| --- | --- |
| Lip | C00 |
| Oral cavity | C02.0-C02.3, C02.8, C02.9, C03, C04, C05.0, C05.3-C05.9 |
| Salivary glands | C07, C08 |
| Oropharynx | C01, C05.1, C05.2, C09, C10 |
| Nasopharynx | C11 |
| Hypopharynx | C12, C13 |
| Pharynx, NOS or overlapping | C14 |
| Nasal sinuses | C30, C31 |
| Larynx | C32 |
| NOS: Not otherwise specified |  |

**Supplementary Table 2:** Distribution of histology codes from the International Classification of Diseases for Oncology, Third Edition (ICD-O-3) by head and neck cancer sites.

| Histology code | Lip  N (%)  N = 972 | Oral cavity  N (%)  N = 1695 | Salivary glands  N (%)  N = 334 | Oropharynx  N (%)  N = 1,150 | Nasopharynx  N (%)  N = 267 | Hypopharynx  N (%)  N = 623 | Pharynx, NOS  N (%)  N = 63 | Nasal sinuses  N (%)  N = 211 | Larynx  N (%)  N = 2,651 | Overall  N (%)  N = 7,966 |
| --- | --- | --- | --- | --- | --- | --- | --- | --- | --- | --- |
| 8000 | 10 (1.03) | 42 (2.48) | 27 (8.08) | 36 (3.13) | 11 (4.12) | 23 (3.69) | 5 (7.94) | 15 (7.11) | 151 (5.70) | 320 (4.02) |
| 8003 | 0 (0.00) | 0 (0.00) | 1 (0.30) | 0 (0.00) | 0 (0.00) | 0 (0.00) | 0 (0.00) | 0 (0.00) | 0 (0.00) | 1 (0.01) |
| 8010 | 3 (0.31) | 17 (1.00) | 30 (8.98) | 17 (1.48) | 25 (9.36) | 12 (1.93) | 3 (4.76) | 3 (1.42) | 21 (0.79) | 131 (1.64) |
| 8012 | 0 (0.00) | 1 (0.06) | 7 (2.10) | 3 (0.26) | 3 (1.12) | 1 (0.16) | 0 (0.00) | 1 (0.47) | 1 (0.04) | 12 (0.21) |
| 8013 | 0 (0.00) | 0 (0.00) | 0 (0.00) | 1 (0.09) | 0 (0.00) | 0 (0.00) | 0 (0.00) | 0 (0.00) | 1 (0.04) | 2 (0.03) |
| 8020 | 0 (0.00) | 2 (0.12) | 7 (2.10) | 3 (0.26) | 40 (14.98) | 4 (0.64) | 0 (0.00) | 8 (3.79) | 4 (0.15) | 68 (0.85) |
| 8021 | 0 (0.00) | 0 (0.00) | 0 (0.00) | 0 (0.00) | 0 (0.00) | 0 (0.00) | 0 (0.00) | 1 (0.47) | 0 (0.00) | 1 (0.01) |
| 8022 | 0 (0.00) | 1 (0.06) | 1 (0.30) | 0 (0.00) | 0 (0.00) | 0 (0.00) | 0 (0.00) | 0 (0.00) | 0 (0.00) | 2 (0.03) |
| 8033 | 0 (0.00) | 2 (0.12) | 0 (0.00) | 0 (0.00) | 0 (0.00) | 2 (0.32) | 1 (1.59) | 1 (0.47) | 6 (0.23) | 12 (0.15) |
| 8034 | 0 (0.00) | 0 (0.00) | 0 (0.00) | 0 (0.00) | 1 (0.37) | 0 (0.00) | 0 (0.00) | 0 (0.00) | 0 (0.00) | 1 (0.01) |
| 8041 | 0 (0.00) | 1 (0.06) | 4 (1.20) | 1 (0.09) | 0 (0.00) | 2 (0.32) | 0 (0.00) | 3 (1.42) | 2 (0.08) | 13 (0.16) |
| 8046 | 0 (0.00) | 0 (0.00) | 1 (0.30) | 0 (0.00) | 0 (0.00) | 0 (0.00) | 0 (0.00) | 0 (0.00) | 0 (0.00) | 1 (0.01) |
| 8051 | 10 (1.03) | 44 (2.60) | 0 (0.00) | 4 (0.35) | 0 (0.00) | 1 (0.16) | 0 (0.00) | 0 (0.00) | 26 (0.98) | 85 (1.07) |
| 8052 | 0 (0.00) | 3 (0.18) | 0 (0.00) | 3 (0.26) | 0 (0.00) | 0 (0.00) | 0 (0.00) | 2 (0.95) | 3 (0.11) | 11 (0.14) |
| 8070 | 822 (84.57) | 1,319 (77.82) | 57 (17.07) | 935 (81.30) | 72 (26.97) | 489 (78.49) | 42 (66.67) | 96 (45.50) | 2,128 (80.27) | 5,960 (74.81) |
| 8071 | 111 (11.42) | 168 (9.91) | 12 (3.59) | 62 (5.39) | 6 (2.25) | 55 (8.83) | 5 (7.94) | 13 (6.16) | 222 (8.37) | 654 (8.21) |
| 8072 | 1 (0.10) | 25 (1.48) | 2 (0.60) | 45 (3.91) | 34 (12.73) | 18 (2.89) | 3 (4.76) | 3 (1.42) | 45 (1.70) | 176 (2.21) |
| 8073 | 0 (0.00) | 1 (0.06) | 0 (0.00) | 0 (0.00) | 2 (0.75) | 0 (0.00) | 0 (0.00) | 1 (0.47) | 0 (0.00) | 4 (0.05) |
| 8074 | 2 (0.21) | 3 (0.18) | 0 (0.00) | 1 (0.09) | 0 (0.00) | 3 (0.48) | 0 (0.00) | 1 (0.47) | 5 (0.19) | 15 (0.19) |
| 8075 | 1 (0.10) | 1 (0.06) | 0 (0.00) | 0 (0.00) | 0 (0.00) | 0 (0.00) | 1 (1.59) | 0 (0.00) | 0 (0.00) | 3 (0.04) |
| 8076 | 8 (0.82) | 4 (0.24) | 0 (0.00) | 0 (0.00) | 0 (0.00) | 0 (0.00) | 0 (0.00) | 0 (0.00) | 7 (0.26) | 19 (0.24) |
| 8082 | 0 (0.00) | 1 (0.06) | 6 (1.80) | 4 (0.35) | 67 (25.09) | 3 (0.48) | 0 (0.00) | 1 (0.47) | 0 (0.00) | 82 (1.03) |
| 8083 | 0 (0.00) | 5 (0.30) | 1 (0.30) | 5 (0.44) | 0 (0.00) | 4 (0.64) | 1 (1.60) | 1 (0.47) | 14 (0.53) | 31 (0.39) |
| 8084 | 0 (0.00) | 0 (0.00) | 2 (0.60) | 1 (0.09) | 0 (0.00) | 2 (0.32) | 0 (0.00) | 0 (0.00) | 0 (0.00) | 5 (0.06) |
| 8085 | 0 (0.00) | 0 (0.00) | 0 (0.00) | 3 (0.26) | 0 (0.00) | 0 (0.00) | 0 (0.00) | 0 (0.00) | 0 (0.00) | 3 (0.04) |
| 8086 | 0 (0.00) | 0 (0.00) | 0 (0.00) | 0 (0.00) | 0 (0.00) | 0 (0.00) | 0 (0.00) | 0 (0.00) | 1 (0.04) | 1 (0.01) |
| 8120 | 0 (0.00) | 0 (0.00) | 0 (0.00) | 0 (0.00) | 1 (0.37) | 0 (0.00) | 0 (0.00) | 2 (0.95) | 0 (0.00) | 3 (0.04) |
| 8121 | 0 (0.00) | 0 (0.00) | 0 (0.00) | 0 (0.00) | 0 (0.00) | 0 (0.00) | 0 (0.00) | 1 (0.47) | 0 (0.00) | 1 (0.01) |
| 8123 | 0 (0.00) | 0 (0.00) | 1 (0.30) | 1 (0.09) | 0 (0.00) | 1 (0.16) | 0 (0.00) | 0 (0.00) | 0 (0.00) | 3 (0.04) |
| 8130 | 0 (0.00) | 0 (0.00) | 0 (0.00) | 0 (0.00) | 0 (0.00) | 0 (0.00) | 0 (0.00) | 1 (0.47) | 0 (0.00) | 1 (0.01) |
| 8140 | 0 (0.00) | 9 (0.53) | 22 (6.59) | 5 (0.44) | 1 (0.37) | 1 (0.16) | 1 (1.59) | 24 (11.37) | 2 (0.08) | 65 (0.82) |
| 8144 | 0 (0.00) | 0 (0.00) | 0 (0.00) | 0 (0.00) | 0 (0.00) | 0 (0.00) | 0 (0.00) | 7 (3.32) | 0 (0.00) | 7 (0.09) |
| 8147 | 0 (0.00) | 0 (0.00) | 5 (1.50) | 0 (0.00) | 0 (0.00) | 0 (0.00) | 0 (0.00) | 1 (0.47) | 0 (0.00) | 6 (0.08) |
| 8200 | 0 (0.00) | 16 (0.94) | 32 (9.58) | 10 (0.87) | 1 (0.37) | 0 (0.00) | 0 (0.00) | 12 (5.69) | 0 (0.00) | 71 (0.89) |
| 8240 | 0 (0.00) | 0 (0.00) | 0 (0.00) | 0 (0.00) | 0 (0.00) | 0 (0.00) | 0 (0.00) | 1 (0.47) | 1 (0.04) | 2 (0.03) |
| 8246 | 0 (0.00) | 0 (0.00) | 0 (0.00) | 0 (0.00) | 0 (0.00) | 2 (0.32) | 0 (0.00) | 1 (0.47) | 2 (0.08) | 5 (0.06) |
| 8249 | 0 (0.00) | 0 (0.00) | 0 (0.00) | 0 (0.00) | 0 (0.00) | 0 (0.00) | 0 (0.00) | 1 (0.47) | 0 (0.00) | 1 (0.01) |
| 8290 | 0 (0.00) | 0 (0.00) | 1 (0.30) | 0 (0.00) | 0 (0.00) | 0 (0.00) | 0 (0.00) | 0 (0.00) | 0 (0.00) | 1 (0.01) |
| 8310 | 0 (0.00) | 1 (0.06) | 0 (0.00) | 0 (0.00) | 0 (0.00) | 0 (0.00) | 0 (0.00) | 1 (0.47) | 0 (0.00) | 2 (0.03) |
| 8410 | 1 (0.10) | 0 (0.00) | 0 (0.00) | 0 (0.00) | 0 (0.00) | 0 (0.00) | 0 (0.00) | 0 (0.00) | 0 (0.00) | 1 (0.01) |
| 8430 | 3 (0.31) | 21 (1.24) | 52 (15.57) | 9 (0.78) | 1 (0.37) | 0 (0.00) | 0 (0.00) | 0 (0.00) | 2 (0.08) | 88 (1.11) |
| 8440 | 0 (0.00) | 1 (0.06) | 1 (0.30) | 0 (0.00) | 0 (0.00) | 0 (0.00) | 0 (0.00) | 0 (0.00) | 0 (0.00) | 2 (0.03) |
| 8480 | 0 (0.00) | 1 (0.06) | 0 (0.00) | 0 (0.00) | 0 (0.00) | 0 (0.00) | 0 (0.00) | 2 (0.95) | 0 (0.00) | 3 (0.04) |
| 8481 | 0 (0.00) | 0 (0.00) | 0 (0.00) | 0 (0.00) | 0 (0.00) | 0 (0.00) | 1 (1.59) | 3 (1.42) | 0 (0.00) | 4 (0.05) |
| 8500 | 0 (0.00) | 0 (0.00) | 16 (4.79) | 0 (0.00) | 0 (0.00) | 0 (0.00) | 0 (0.00) | 0 (0.00) | 0 (0.00) | 16 (0.20) |
| 8525 | 0 (0.00) | 1 (0.06) | 1 (0.30) | 0 (0.00) | 0 (0.00) | 0 (0.00) | 0 (0.00) | 0 (0.00) | 0 (0.00) | 2 (0.03) |
| 8550 | 0 (0.00) | 1 (0.06) | 38 (11.38) | 0 (0.00) | 0 (0.00) | 0 (0.00) | 0 (0.00) | 1 (0.47) | 0 (0.00) | 40 (0.50) |
| 8560 | 0 (0.00) | 3 (0.18) | 1 (0.30) | 1 (0.09) | 1 (0.37) | 0 (0.00) | 0 (0.00) | 0 (0.00) | 7 (0.26) | 13 (0.16) |
| 8562 | 0 (0.00) | 1 (0.06) | 6 (1.80) | 0 (0.00) | 1 (0.37) | 0 (0.00) | 0 (0.00) | 2 (0.95) | 0 (0.00) | 10 (0.13) |
| 8711 | 0 (0.00) | 0 (0.00) | 0 (0.00) | 0 (0.00) | 0 (0.00) | 0 (0.00) | 0 (0.00) | 1 (0.47) | 0 (0.00) | 1 (0.01) |
